# Comparison of pilot tone-triggered and electrocardiogram-triggered cardiac magnetic resonance imaging: a prospective clinical feasibility study

**DOI:** 10.1016/j.jocmr.2025.101925

**Published:** 2025-06-19

**Authors:** Xianling Qian, Yali Wu, Peter Speier, Caixia Fu, Yunzhu Wu, Lude Cheng, Yinyin Chen, Shiyu Wang, Caizhong Chen, Kai Liu, Ling Chen, Hang Jin, Mengsu Zeng

**Affiliations:** a Department of Radiology, Zhongshan Hospital, Fudan University, Shanghai, China; b Shanghai Institute of Medical Imaging, Shanghai, China; c Research & Clinical Translation, Magnetic Resonance, Siemens Healthineers AG, Erlangen, Germany; d MR Application Development, Siemens Shenzhen Magnetic Resonance Ltd., Shenzhen, China; e MR Research Collaboration, Siemens Healthineers, Shanghai, China; f MR Application, Siemens Healthineers, Shanghai, China

**Keywords:** Cardiac magnetic resonance, Pilot tone, Electrocardiogram

## Abstract

**Background:**

Electrocardiogram (ECG)-triggered cardiovascular magnetic resonance (CMR) can be challenging in patients with ECG unreliability. Pilot tone (PT)-triggered CMR may offer a reliable alternative.

**Purpose:**

To evaluate the feasibility of PT-triggered CMR and compare its performance with ECG-triggered imaging across various sequences in patients with common cardiovascular diseases.

**Methods:**

This prospective study included 50 participants (26 males, 24 females; mean age 46.0 ± 19.0 y), including 15 with normal CMR findings and 35 with various cardiovascular diseases. All participants underwent both PT-triggered and ECG-triggered CMR on a 3T MRI system. Imaging included T2-weighted imaging (T2WI), T1-mapping, T2-mapping, cine, late gadolinium enhancement (LGE), and post-contrast T1-mapping sequences. Image quality and quantitative measurements were evaluated, including T2WI signal intensity, native T1-mapping, T2-mapping, and extracellular volume fraction (ECV) values, and comparative signal-to-noise ratio (compSNR) and comparative contrast-to-noise ratio (compCNR) of cine and LGE images, left/right ventricular function. Inter-reader agreement was evaluated using the intraclass correlation coefficient (ICC). Comparisons between the two methods were performed using paired *t*-test or the Wilcoxon signed-rank test.

**Results:**

No significant differences were observed in scanning times (*p *= .253–.864) or image quality (ICC: .589–1.000, *p *= .057–1.000) between PT- and ECG-triggered scans and images. Quantitative assessments showed good to excellent consistency (ICC = .843–.987). While PT-triggered LGE images showed higher compCNR (14.14 ± 7.68 vs. 13.24 ± 7.52, *p *= .016), other quantitative parameters showed no significant differences between PT- and ECG-triggered images. Six participants with hypertrophic cardiomyopathy or heart valve disease experienced false R-wave triggering during ECG gating, leading to motion artifacts, which were not visible in PT-triggered images.

**Conclusion:**

PT-triggered cardiac MRI provides comparable image quality and quantitative assessments to ECG-triggered sequences and may offer advantages in minimizing motion artifacts, particularly in patients with conditions affecting ECG reliability, making it a promising alternative for cardiac MRI synchronization.

## Graphical abstract

**Figure:**
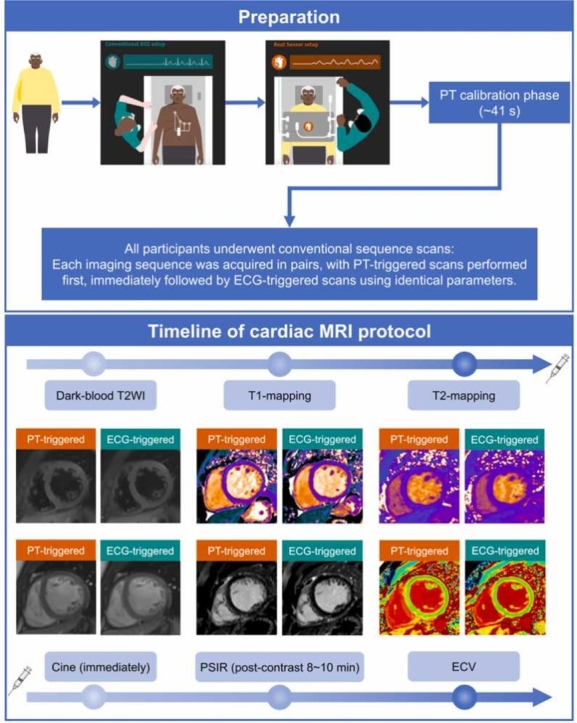

## 1 Introduction

Cardiovascular magnetic resonance imaging (CMR) relies on precise synchronization with the cardiac cycle to minimize artifacts and ensure high-quality imaging [1], [2]. Conventionally, the electrocardiogram (ECG) R-wave serves as the trigger signal for image acquisition, aligning scans with cardiac motion [3], [4]. However, ECG-based synchronization has notable limitations. Gradient and radiofrequency (RF) interference as well as the magnetohydrodynamic (MHD) effect within the MRI scanner can distort ECG signals, complicating R-wave detection [4], [5], [6]. Additionally, skin preparation is required, and ECG hardware is prone to heating, potentially causing burns resulting from induction of high voltages, while sweating can lead to electrode detachment, prolonging scan times and increasing motion-related artifacts [7], [8], [9], [10]. Moreover, ECG setup requires meticulous electrode adjustments, potentially causing discomfort [11], and potential privacy concerns for female patients. These challenges highlight the need for alternative synchronization methods that address the drawbacks of ECG-triggered imaging.

Several synchronization strategies including pulse wave [12], self-gating technique [13], Doppler ultrasound [14], [15], [16], and camera-based photoplethysmography [17], have been explored to address these limitations. In addition, recent advancements such as real-time cardiac MRI, free-running imaging, and MR multitasking aim to eliminate the need for gating altogether [18], [19], [20]. However, these techniques have not yet been widely adopted in routine clinical settings due to challenges such as limited spatial and temporal resolution, extended reconstruction times, and the requirement for specialized software or hardware. Recently, the Pilot Tone (PT) technology has emerged as a promising alternative [21]. PT navigation is an MR image-independent motion detection method introduced by Speier et al. [22], [23], [24], [25]. This system employs a small loop antenna integrated into the body coil array to generate a continuous-wave RF signal at a frequency outside the MR imaging signal band, avoiding interference with image acquisition while remaining within the receiver bandwidth. The generated signal, modulated by underlying motion, is captured by all active receiver coils, enabling the extraction of respiratory curves and cardiac triggers comparable to conventional ECG gating during MRI acquisition. PT offers a higher sampling rate independent of image acquisition, making it a valuable alternative to MR data-driven self-gating approaches [26], [27].

This study aimed to evaluate the clinical feasibility of PT-triggered cardiac MRI and to comprehensively compare its performance across conventional sequences, including T2-weighted imaging (T2WI), modified Look-Locker inverstion recovery (MOLLI) T1-mapping, T2-mapping, cine imaging, late gadolinium enhancement (LGE), and extracellular volume (ECV), with standard ECG synchronization in the context of common cardiovascular diseases.

## 2 Materials and methods

### 2.1 Study participants

This prospective study was approved by the hospital’s ethics committee (Approval No. B2023–371), and written informed consent was obtained from all participants prior to enrollment. The inclusion criterion was a clinical indication for CMR Exclusion criteria included (a) pregnancy, (b) metallic implants or devices contraindicated for magnetic resonance imaging (MRI), (c) known gadolinium-based contrast agent allergies, (d) estimated creatinine clearance ≤30 mL/min, or (e) severe claustrophobia. From May 2024 to August 2024, 50 participants were recruited, 47 of whom underwent gadolinium-enhanced MRI, while 3 underwent non-contrast scans. Among these, 15 participants exhibited no diagnostic findings on CMR, while the remaining 35 were diagnosed with a variety of cardiovascular diseases.

### 2.2 Study design and CMR protocol

**Fig. 1:**
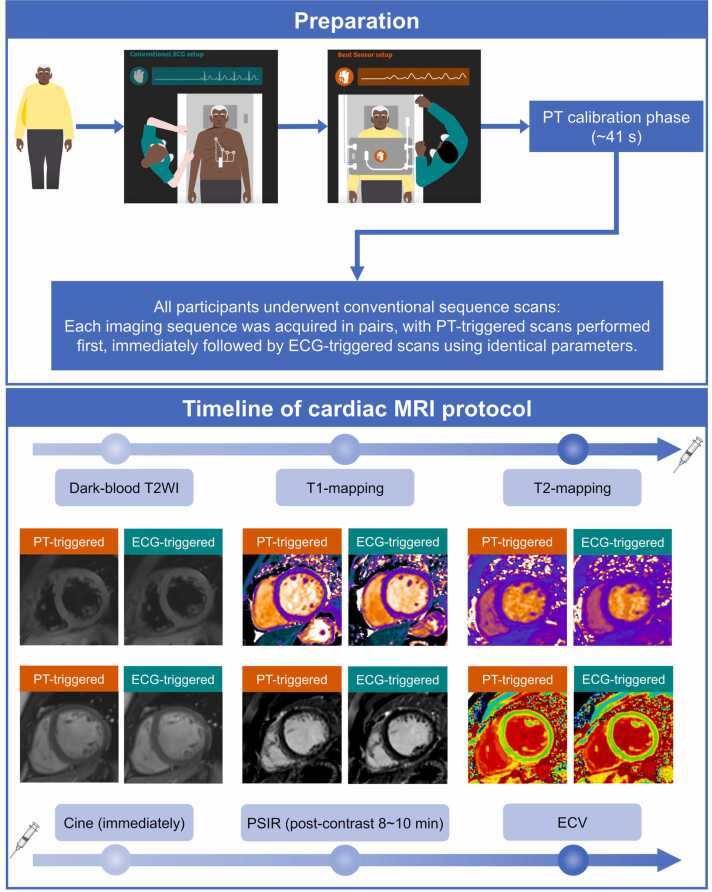
Flowchart of the prospective comparative study. *ECG* electrocardiogram, *T2WI* T2-weighted imaging, *LGE* late gadolinium enhancement, *ECV* extracellular volume, *PT* pilot tone

All participants underwent both PT-triggered and ECG-triggered CMR scans on a 3T MRI system (MAGNETOM Seara, Siemens Healthineers, Erlangen, Germany), which features a 70 cm bore diameter, a maximum gradient amplitude of 78 mT/m, and a maximum slew rate of 346 T/m/s. Imaging was performed using a 12-channel BioMatrix Body array coil with integrated PT generator [22], [23], [24], [25], alongside an integrated 24-channel spine coil as receivers. ECG leads were applied before positioning the body array coil to enable ECG-triggered scans as a reference, and no retraining or alternative trigger mode was available for optimization. The PT-based triggering system detects cardiac motion by capturing modulations in a continuous RF signal and extracting cardiac triggers accordingly [22], [23], [24], [25]. The PT-based triggering system required an initial calibration (learning) phase of approximately 41 s before image acquisition [28]. This calibration remained stable throughout the examination, provided that the active receiver coils remained in their initial position and the participant did not undergo significant changes in body position. No recalibration was necessary, and no calibration failures were encountered among the 50 participants in this study. Examination protocols included turbo spin echo (TSE) dark-blood T2WI, MOLLI T1-mapping, T2-mapping, followed by contrast injection, cine, post-contrast breath-hold phase-sensitive inversion recovery (PSIR) at 8–10 min, and post-contrast T1-mapping. All sequences were performed sequentially under PT and ECG triggering with identical parameters, and ECV images were post-processed. Fig. 1 shows the study design, with detailed protocol parameters provided in Table 1.

**Table 1:** CMR acquisition parameters.

Variables	T2WI		Native T1-mapping		T2-mapping	Cine	PSIR	Post T1-mapping	
Sequence type	TSE		TrueFISP		GRE	TrueFISP	GRE	TrueFISP	
R-R interval (ms)	> 700	< 700	> 700	< 700	/	/	/	> 700	< 700
Trigger delay after R-wave for ECG scan (ms)*	0	0	400	407	596	/	0	320	335
No. of trigger pulse	2	2	1	1	1	/	2	1	1
Acquisition window (ms)	800	650	700	700	804	/	750	705	700
Echo time (ms)	42	70	1.12	1.01	1.32	1.53	1.95	1.12	1.01
Repetition time (ms)	800	600	280.56	263.88	207.4	34.8	750	360.56	341.44
Flip angle (degree)	180	180	35	35	12	80	20	35	35
Field of view (mm^2^)	293 × 360	293 × 360	307 × 360	307 × 360	289 × 360	264 × 340	290 × 360	307 × 360	307 × 360
Acquisition matrix	166 × 256	208 × 256	144 × 256	132 × 192	116 × 192	122 × 224	152 × 256	144 × 256	128 × 192
Spatial resolution (mm^2^)	1.4 × 1.4	1.4 × 1.4	1.4 × 1.4	1.9 × 1.9	1.9 × 1.9	1.5 × 1.5	1.4 × 1.4	1.4 × 1.4	1.9 × 1.9
Echo spacing (ms)	5.27	4.12	2.7	2.44	3.15	3.48	5.15	2.7	2.44
Bandwidth (Hz/pixel)	849	849	1085	1085	1184	970	287	1085	1085
Section thickness (mm)	6	5	8	8	8	8	8	8	8
Gap (%)	50	50	20	20	20	20	20	20	20

*T2WI* T2-weighted imaging, *PSIR* phase-sensitive inversion recovery, *TSE* turbo spin echo, *TrueFISP* true fast imaging with steady-state precession, *GRE* gradient recalled echo, *ECG* electrocardiogram.

All sequences used GRAPPA parallel imaging (acceleration factor = 2).

Prescan-based surface coil normalization was applied.

Native T1 mapping: MOLLI 5(3)3; post-contrast T1 mapping: MOLLI 4(1)3(1)2.

T2mapping: T2 preparation durations were 0 ms, 35 ms, 55 ms.

* The timing of cardiac triggers detected by the Pilot Tone (PT) is about 180 ms delayed with respect to the R-wave detected by ECG triggering. This delay is automatically compensated for by the scanner software: when switching from ECG to PT triggering, the system reduces the trigger delay accordingly, except in the case of the TSE dark-blood T2WI sequence, where the fixed timing of the double-inversion preparation makes automatic compensation less feasible

Data are presented as nominal values for each cardiac MR sequence.

### 2.3 Qualitative analysis

The qualitative assessment of T2WI, native T1-mapping, T2-mapping, cine, LGE, and post-T1-mapping images was independently conducted by two blinded radiologists, S.Y.W. and Y.Y.C., with 10 and 15 y of experience in CMR diagnostics, respectively. Image quality was evaluated for the basal, mid-ventricular, and apical sections using a five-point Likert scale as follows: 1) poor, non-diagnostic; 2) fair, with noticeable motion artifacts or distortion but partially diagnostic; 3) adequate, with moderate motion artifacts or distortion yet diagnostic; 4) good, with mild motion artifacts or distortion; and 5) excellent, with minimal to no motion artifacts or distortion [29]. Before initiating the assessment, both readers participated in a training session to familiarize themselves with the classification system.

### 2.4 Quantitative analysis

Quantitative analysis was independently performed by two radiologists, X.L.Q. and W.Y.L., with 5 and 3 years of cardiac MRI experience, respectively, using Circle Cardiovascular Imaging software (version 5.17.0, Circle Cardiovascular Imaging Inc., Calgary, Canada). Signal intensity (SI) measurements were performed at three short-axis slices as follows: basal, mid-ventricular, and apical.

For T2WI analysis, four myocardial regions of interest (ROIs) were drawn in the anterior, septal, inferior, and lateral walls at each slice, avoiding areas with visible pathology. A corresponding ROI was placed in the adjacent pectoralis major muscle on the same slice, avoiding artifacts such as wraparound. The average myocardial SI was divided by skeletal muscle SI to derive SI_myo_/SI_SM_ for each slice, and the average across the three slices was used for final analysis.

For cine and LGE images, ROIs were placed on the same three short-axis slices. Four myocardial ROIs were drawn per slice, and a single large ROI was placed in the left ventricular blood pool, avoiding papillary muscles and chordae tendineae. Image noise was estimated by placing three ROIs in air outside the body on the same slice. For each slice, the mean SI of the four myocardial ROIs, the mean SI of the blood pool ROI, and the mean of the SI standard deviations (SDs) from the three background air ROIs were calculated and then used in the corresponding comparative signal-to-noise ratio (compSNR) and comparative contrast-to-noise ratio (compCNR) equations, as shown below [30], [31]. All values were then averaged across the three slices. In participants with LGE, the LGE compCNR measurement was performed on the slice with the most prominent scar by placing a large ROI within the LGE lesion. All ROIs were initially drawn on PT-triggered images and then directly copied to ECG-triggered images in the Viewer module, ensuring spatially identical ROI placement for all paired comparisons.

Using T1 and T2 mapping module, native T1, T2, and post-contrast T1 values were automatically derived from T1 and T2 maps following contour delineation of basal, mid-ventricular, and apical sections, with ECV maps calculated using hematocrit data. Measurements followed the AHA 16-segment and global models.

Left and right ventricular function was evaluated by tracing endocardial and epicardial borders on short-axis cine images at end-diastolic and end-systolic phases, with manual adjustments as needed. For participants with LGE, myocardial fibrosis was semi-automatically identified based on SI thresholds: 2–3 SDs above remote myocardium for non-ischemic and 5 SDs for ischemic cardiomyopathy. Minor manual adjustments were made as needed to refine region boundaries, exclude artifacts, or correct misclassified areas for accurate LGE mass quantification.

$\mathrm{Cine}\;{\mathrm{compSNR}}_{\mathrm{blood}}=\frac{{\mathrm{mean}}_{\mathrm{SI}}\;\mathrm{of}\;\mathrm{blood}}{{1.5\times \mathrm{SD}}_{\mathrm{SI}}\;\mathrm{of}\;\mathrm{air}\;\mathrm{outside}\;\mathrm{the}\;\mathrm{body}}$

$\mathrm{Cine}\;{\mathrm{compSNR}}_{\mathrm{myo}}=\frac{{\mathrm{mean}}_{\mathrm{SI}}\;\mathrm{of}\;\mathrm{myocardium}\;}{{1.5\times \mathrm{SD}}_{\mathrm{SI}}\;\mathrm{of}\;\mathrm{air}\;\mathrm{outside}\;\mathrm{the}\;\mathrm{body}}$

$\mathrm{Cine}\;\mathrm{compCNR}=\frac{{\mathrm{mean}}_{\mathrm{SI}}\;\mathrm{of}\;\mathrm{blood}-{\mathrm{mean}}_{\mathrm{SI}}\;\mathrm{of}\;\mathrm{myocardium}\;}{{1.5\times \mathrm{SD}}_{\mathrm{SI}}\;\mathrm{of}\;\mathrm{air}\;\mathrm{outside}\;\mathrm{the}\;\mathrm{body}}$

$\mathrm{LGE}\;\mathrm{compSNR}=\frac{{\mathrm{mean}}_{\mathrm{SI}}\;\mathrm{of}\;\mathrm{remote}\;\mathrm{myocardium}\;}{{1.5\times \mathrm{SD}}_{\mathrm{SI}}\;\mathrm{of}\;\mathrm{air}\;\mathrm{outside}\;\mathrm{the}\;\mathrm{body}}$

$L\mathrm{GE}\;\mathrm{comp}\mathrm{CNR}=\frac{{\mathrm{mean}}_{\mathrm{SI}}\;\mathrm{of}\;s\mathrm{car}-{m\mathrm{ean}}_{SI}\;\mathrm{of}\;\mathrm{remote}\;\mathrm{myocardium}}{1.5\times {\mathrm{SD}}_{SI}\;\mathrm{of}\;\mathrm{air}\;\mathrm{outside}\;\mathrm{the}\;\mathrm{body}}\;(\mathrm{with}\;\mathrm{LGE})$

$L\mathrm{GE}\;\mathrm{comp}\mathrm{CNR}=\frac{{\mathrm{mean}}_{\mathrm{SI}}\;\mathrm{of}\;b\mathrm{lood}-{m\mathrm{ean}}_{SI}\;\mathrm{of}\;\mathrm{remote}\;\mathrm{myocardium}}{1.5\times {\mathrm{SD}}_{SI}\;\mathrm{of}\;\mathrm{air}\;\mathrm{outside}\;\mathrm{the}\;\mathrm{body}}\;(\mathrm{witho}\mathrm{ut}\;\mathrm{LGE})$

### 2.5 Statistical analysis

Data normality was assessed using the Shapiro-Wilk test. Normally distributed data are presented as mean ± SD and compared using the Student’s *t*-test, while non-normally distributed data are shown as median and interquartile range (IQR) and compared using the Mann-Whitney *U* test. Categorical data are presented as frequencies and percentages, with the Chi-square test or Fisher’s exact test used for comparison. Inter-reader agreement was evaluated using Bland-Altman plots and the intraclass correlation coefficient (ICC), categorized as poor (<0.50), moderate (0.50–0.75), good (0.75–0.90), and excellent (>0.90) [32], [33]. While ICC assumes normality to some extent, the results should be interpreted as approximations when applied to non-normally distributed data. All quantitative and qualitative measurements were averaged from both readers’ assessments. Comparative analysis between paired samples was conducted using the paired *t*-test for normally distributed data or the Wilcoxon signed-rank test for non-normally distributed data. Statistical analyses were performed using R software (version 4.1.1). Two-sided tests were applied, with a *p* <.05 considered statistically significant.

## 3 Results

### 3.1 Baseline characteristics and acquisition time

The study included 50 participants, comprising 26 (52%) males and 24 (48%) females. The mean age was 46.0 ± 19.0 y, with a range of 14–84 y. The mean weight was 65.3 ± 16.3 kg, with a range of 40.0 to 102.5 kg. The baseline demographic characteristics and CMR findings are presented in Table 2.

**Table 2:** Baseline characteristics of participants.

Baselines	All participants (*n* = 50)
Age (y)^a^	46.04 ± 19.01
Gender^b^	
Male	26 (52%)
Female	24 (48%)
Height (cm)^a^	168.00 ± 9.87
Weight (kg)^a^	65.28 ± 16.26
BSA (m^2^)^a^	1.83 ± 0.24
HR (bpm)^a^	71.42 ± 15.54
Diagnosis^b^	
Normal	15 (30%)
HCM	7 (14%)
HVD, cardiac dilatation	6 (12%)
DCM	5 (10%)
Cardiac dilatation	3 (6%)
Pericardial effusion	3 (6%)
ICM	2 (4%)
Myocarditis	2 (4%)
NVM	2 (4%)
Left ventricular lipoma	2 (4%)
Cardiac amyloidosis	1 (2%)
Hypertensive cardiopathy	1 (2%)
Myocardial fibrosis	1 (2%)

*BSA* body surface area, *HR* heart rate, *HCM* hypertrophic cardiomyopathy, *HVD* heart valve disease, *DCM* dilated cardiomyopathy, *ICM* ischemic cardiomyopathy, *NVM* noncompaction of ventricular myocardium

a Data are means ± standard deviations

b Data are the number of participants with the percentage in parentheses

The ranges of time intervals between gadolinium injection and sequence acquisition were as follows: PT-triggered LGE: 8.1–9.9 min; ECG-triggered LGE: 10.4–16.9 min; PT-triggered post-contrast T1 mapping: 13.07–19.57 min; ECG-triggered post-contrast T1 mapping: 15.43–22.32 min. No significant differences were observed in single-section scanning times for conventional sequences, whether triggered by PT or ECG. These sequences included T2WI (*p* =.612), native T1-mapping (*p* =.714), T2-mapping (*p* =.253), cine imaging (*p* =.637), LGE (*p* =.864), and post T1-mapping (*p* =.750), as detailed in Table 3.

**Table 3:** Comparison of single-section scanning times triggered by PT and ECG.

Sequences	PT (s)	ECG (s)	*p*-value
T2WI^a^	8.32 (7.15 ∼ 9.33)	8.39 (7.41 ∼ 10.11)	.612
Native T1-mapping^a^	16.50 (14.00 ∼ 19.00)	16.00 (14.00 ∼ 19.00)	.714
T2-mapping^a^	15.00 (12.60 ∼ 16.40)	15.00 (12.00 ∼ 16.00)	.253
Cine^b^	4.85±1.02	4.75±1.01	.637
LGE^a^	8.17 (6.58 ∼10.35)	8.06 (6.78 ∼ 10.83)	.864
Post T1-mapping^a^	17.00 (14.67 ∼ 19.00)	17.00 (14.00 ∼ 19.00)	.750

*PT* Pilot Tone, *ECG* electrocardiogram, *T2WI* T2-weighted imaging, *LGE* late gadolinium enhancement

a Data are medians with interquartile ranges in parentheses

b Data are means ± standard deviations

### 3.2 Qualitative comparison between PT- and ECG-triggered images

The two readers exhibited good to excellent agreement in assessing the image quality of PT- and ECG-triggered images (Table S1). As presented in Table 4, the T2WI, native T1-mapping, T2-mapping, cine imaging, LGE, and post T1-mapping images triggered by both PT and ECG demonstrated ICCs ranging from moderate to excellent for image quality (ICC:.589–1.000). No significant differences were observed in image quality Likert scores between PT- and ECG-triggered images (*p* =.057–1.000).

**Table 4:** Comparison of image quality Likert scores between PT- and ECG-triggered images.

Sequences	SAX sections	PT	ECG	ICC	*p*-value
T2WI	Basal	4 (3 ∼ 5)	5 (4 ∼ 5)	.611	.059
	Mid	4.5 (4 ∼ 5)	5 (4 ∼ 5)	.622	.562
	Apical	5 (4.5 ∼ 5)	5 (4.125 ∼ 5)	.675	.313
Native T1-mapping	Basal	5 (5 ∼ 5)	5 (5 ∼ 5)	.860	.414
	Mid	5 (5 ∼ 5)	5 (5 ∼ 5)	.765	.785
	Apical	5 (5 ∼ 5)	5 (5 ∼ 5)	.884	.317
T2-mapping	Basal	5 (5 ∼ 5)	5 (5 ∼ 5)	/	1.000
	Mid	5 (5 ∼ 5)	5 (5 ∼ 5)	/	.119
	Apical	5 (5 ∼ 5)	5 (5 ∼ 5)	/	.317
Cine	Basal	5 (4 ∼ 5)	5 (4 ∼ 5)	.826	.057
	Mid	4.5 (4 ∼ 5)	4.5 (4 ∼ 5)	.878	.522
	Apical	5 (4 ∼ 5)	5 (4 ∼ 5)	.960	.194
LGE	Basal	5 (4 ∼ 5)	5 (4 ∼ 5)	.675	.100
	Mid	5 (4.375 ∼ 5)	5 (4 ∼ 5)	.589	.731
	Apical	5 (4.5 ∼ 5)	5 (4.5 ∼ 5)	.701	1.000
Post T1-mapping	Basal	5 (5 ∼ 5)	5 (5 ∼ 5)	1.000	1.000
	Mid	5 (5 ∼ 5)	5 (5 ∼ 5)	.796	.317
	Apical	5 (5 ∼ 5)	5 (5 ∼ 5)	1.000	1.000

*PT* Pilot Tone, *ECG* electrocardiogram, *SAX* short axis, *ICC* intraclass correlation coefficient, *T2WI* T2-weighted imaging, *LGE* late gadolinium enhancement

Data are medians with interquartile ranges in parentheses. The ICC value of 1.000 may reflect the limited granularity of the 5-point Likert scale rather than true perfect agreement.

### 3.3 Quantitative comparison between PT- and ECG-triggered images

**Fig. 2:**
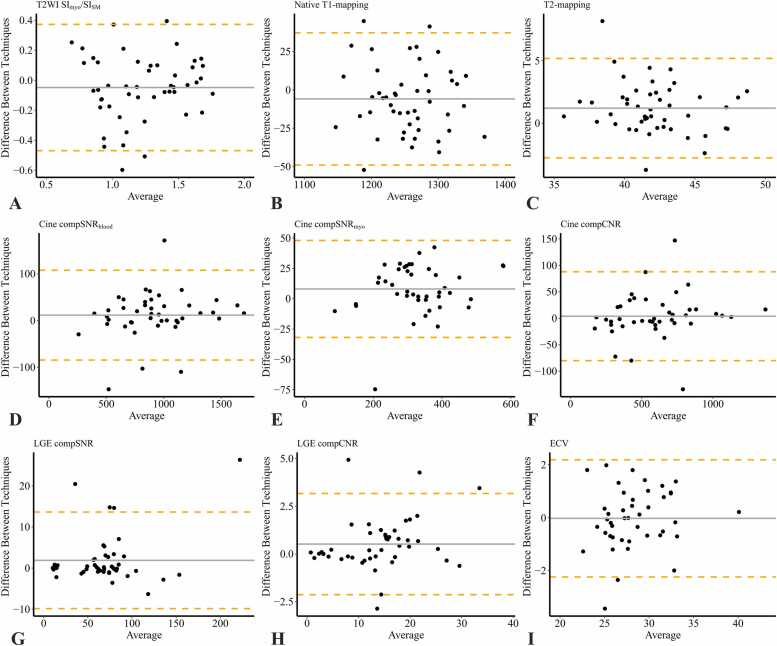
Bland-Altman plots demonstrate the agreement between quantitative parameters derived from PT-triggered and ECG-triggered images. These parameters include the ratio of T2WI signal intensity of myocardium to skeletal muscle (SI_myo_/SI_SM_), native T1-mapping value, T2-mapping value, compSNR and compCNR for cine and LGE images, as well as ECV. *PT* Pilot Tone, *ECG* electrocardiogram, *T2WI* T2-weighted imaging, *compSNR* comparative signal-to-noise ratio, *compCNR* comparative contrast-to-noise ratio, *LGE* late gadolinium enhancement, *ECV* extracellular volume

The two readers demonstrated excellent consistency in the quantitative evaluation of PT-triggered images (Fig. S1 and Table S2) and ECG-triggered images (Fig. S2 and Table S3). As presented in Table 5 and Fig. 2, the consistency between PT- and ECG-triggered images for quantitative evaluation, based on the American Heart Association (AHA) 16-segment model and the whole-heart model, ranged from moderate to excellent.

**Table 5:** Intraclass correlation coefficient of quantitative assessment between PT- and ECG-triggered images.

AHA 16- segment model	T2WI SI_myo_/SI_SM_	Native T1-mapping	T2-mapping	Cine compSNR_blood_	Cine compSNR_myo_	Cine compCNR	LGE compSNR	LGE compCNR	ECV
1	.804	.917	.792	.983	.963	.977	.987	.972	.966
2	.767	.906	.735						.919
3	.780	.887	.798						.931
4	.767	.894	.774						.957
5	.598	.830	.749						.982
6	.615	.883	.815						.901
7	.878	.918	.652						.946
8	.874	.962	.882						.949
9	.898	.918	.880						.943
10	.834	.925	.762						.923
11	.839	.918	.794						.943
12	.854	.934	.876						.936
13	.820	.911	.794						.966
14	.797	.915	.852						.946
15	.823	.921	.612						.982
16	.885	.885	.759						.988
Global	.864	.958	.843						.973

*PT* Pilot Tone, *ECG* electrocardiogram, *AHA* American Heart Association, *T2WI* T2-weighted imaging, *SI* signal intensity, *myo* myocardium, *SM* skeletal muscle, *compSNR* comparative signal-to-noise ratio, *compCNR* comparative contrast-to-noise ratio, *LGE* late gadolinium enhancement, *ECV* extracellular volume

Data are intraclass correlation coefficients (ICC) representing the agreement between PT- and ECG-triggered image measurements.

**Fig. 3:**
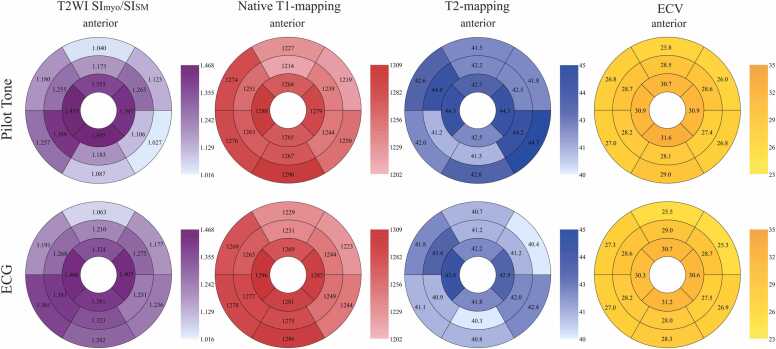
AHA 16-segment models of the ratio of T2WI signal intensity of myocardium to skeletal muscle (SI_myo_/SI_SM_), native T1-mapping value (ms), T2-mapping value (ms), and ECV (%) based on Pilot Tone-triggered and ECG-triggered images. *ECV* extracellular volume, *ECG* electrocardiogram

As shown in Table 5, Table 6, the whole-heart T2WI SI_myo_/SI_SM_ ratios measured on PT- and ECG-triggered images demonstrated good consistency (ICC = .864) with no statistically significant difference (PT: 1.19 [IQR: 0.89–1.47], ECG: 1.25 ± 0.30; *p *= .122). Similar finding was observed for T2-mapping (PT: 42.71 ± 2.88 ms, ECG: 41.50 ± 3.25 ms; ICC = .843, *p *= .773). The whole-heart native T1-mapping measured on PT- and ECG-triggered images demonstrated excellent consistency (ICC = .958) and no statistically significant difference (PT: 1255 ± 56 ms, ECG: 1261 ± 57 ms; *p *= .067). Similar finding was also observed for ECV (PT: 27.63% [IQR: 25.50–30.92], ECG: 27.67% [IQR: 25.99–30.88]; ICC = .973, *p *= .885). The AHA 16-segment models for these parameters triggered by PT and ECG are shown in Fig. 3.

**Table 6:** Comparison of quantitative assessment between PT- and ECG-triggered images.

	PT	ECG	*p*-value
T2WI SI_myo_/SI_SM_	1.19 (0.89 ∼ 1.47)	1.25 ± 0.30	.122
Native T1-mapping (ms)	1254.90 ± 55.84	1260.79 ± 56.74	.067
T2-mapping (ms)	42.71 ± 2.88	41.50 ± 3.25	.773
Cine compSNR_blood_	925.68 ± 331.22	899.04 ± 323.73	.087
Cine compSNR_myo_	332.31 ± 103.19	315.52 ± 98.73	.144
Cine compCNR	598.15 ± 269.04	587.17 ± 266.16	.638
LGE compSNR	70.86 (49.25 ∼ 82.34)	68.16 (48.66 ∼ 78.61)	.232
LGE compCNR	14.14 ± 7.68	13.24 ± 7.52	.016
ECV (%)	27.63 (25.50 ∼ 30.92)	27.67 (25.99 ∼ 30.88)	.885

For non-normally distributed data, the values are presented as the median with the interquartile range in parentheses. For normally distributed data, the values are presented as the mean ± standard deviation.

*PT* Pilot Tone, *ECG* electrocardiogram, *T2WI* T2-weighted imaging, *SI* signal intensity, *myo* myocardium, *SM* skeletal muscle, *compSNR* comparative signal-to-noise ratio, *compCNR* comparative contrast-to-noise ratio, *LGE* late gadolinium enhancement, *ECV* extracellular volume

Cine-compSNR_blood_, Cine-compSNR_myo_, Cine-compCNR, LGE-compSNR, and LGE-compCNR with PT and ECG demonstrated excellent consistency (ICC = .963–.987). Compared to ECG-triggered images, PT-triggered cine images exhibited similar compSNR and compCNR values (*p *= .087–.638). Additionally, PT-triggered LGE-compCNR was higher than ECG-triggered values (14.14 ± 7.68 vs. 13.24 ± 7.52, *p *= .016), whereas LGE-compSNR showed no significant difference (*p *= .232).

**Fig. 4:**
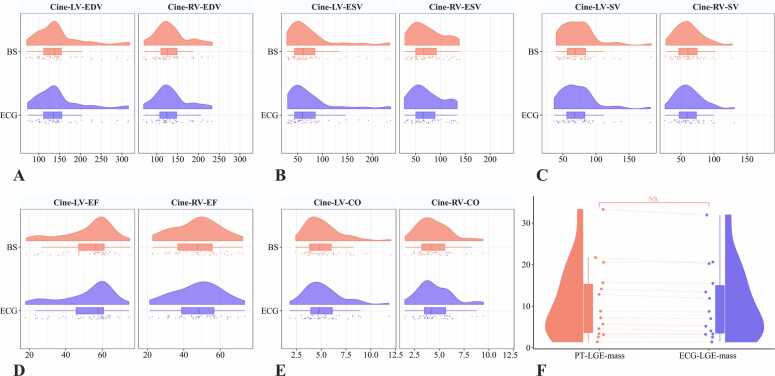
Raincloud plots illustrate the agreement in left and right ventricular function parameters, including EDV (mL), ESV (mL), SV (mL), EF (%), and CO (L/min), as well as LGE mass (g), between PT-triggered and ECG-triggered images. *EDV* end-diastolic volume, *ESV* end-systolic volume, *SV* stroke volume, *EF* ejection fraction, *CO* cardiac output, *LGE* late gadolinium enhancement, *PT* Pilot Tone, *ECG* electrocardiogram

The two readers demonstrated excellent consistency in evaluating quantitative LV and RV cardiac function and LGE mass on PT- and ECG-triggered cine and LGE images (Table S4). As illustrated in Fig. 4, excellent agreement was observed between PT- and ECG-triggered images for these parameters, with no statistically significant differences (*p *= .052–.800) (Table S5).

### 3.4 PT-triggered images when R-wave triggering is incorrect

**Fig. 5:**
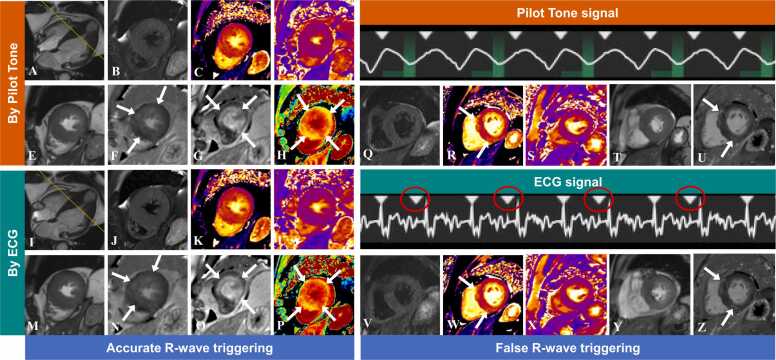
Examples comparing CMR images triggered by PT and ECG, with and without ECG distortion. (**A-P**) PT- and ECG-triggered images with accurate R-wave triggering from a 67-year-old male participant with HCM. The image quality of conventional sequences triggered by PT was comparable to that of ECG-triggered images with accurate R-wave detection. Both PT- and ECG-triggered LGE images, as well as post-processed ECV images, exhibited similar high quality, clearly visualizing the extent of myocardial fibrosis (white arrow). (**Q-Z**) PT- and ECG-triggered images with false R-wave triggering from a 70-year-old female participant with HCM. While both PT- and ECG-triggered T1-mapping and LGE images showed good image quality and clearly depicted myocardial fibrosis (white arrow), ECG-triggered T2WI (**V**) and cine (**Y**) images exhibited motion artifacts and signal loss due to R-wave misgating (red circles), which were absent in PT-triggered images (**Q** and **T**). *PT* Pilot Tone, *ECG* electrocardiogram, *HCM* hypertrophic cardiomyopathy, *LGE* late gadolinium enhancement, *ECV* extracellular volume, T2WI T2-weighted imaging

Six participants experienced interference in ECG signals from magnetic fields during scanning, leading to incorrect recognition or failure to detect R-waves. Fig. 5**A-P** illustrates PT- and ECG-triggered images from a 67-year-old male participant with hypertrophic cardiomyopathy (HCM). The image quality of images triggered by PT was comparable to that of ECG-triggered images with accurate R-wave detection. Both PT-triggered and ECG-triggered LGE images and post-processed ECV images had good and similar image quality, clearly depicting the extent of myocardial fibrosis. Fig. 5**Q-Z** illustrates images from a 70-year-old female participant with HCM experiencing false R-wave triggering during ECG gating. Although both PT-triggered and ECG-triggered T1-mapping and LGE images exhibited good and similar image quality and could clearly depict myocardial fibrosis, ECG-triggered T2WI and cine images displayed blur artifacts and signal loss due to R-wave misgating, which were absent in PT-triggered images.

## 4 Discussion

In this study, we prospectively evaluated the clinical feasibility and stability of PT technology, based on PT navigation, by comparing it with conventional ECG gating. We found that the acquisition time for all conventional sequences triggered by PT was comparable to that of ECG-triggered sequences. The image quality of T2WI, native T1-mapping, T2-mapping, cine, LGE, and post-T1-mapping images acquired with PT was equivalent to that of ECG-triggered images across basal, mid-ventricular, and apical sections. Most quantitative parameters from PT-triggered images were comparable to those of ECG-triggered images. Additionally, six participants in our study experienced false R-wave triggering, resulting in blur artifacts and signal loss, particularly in T2WI and cine images, while PT successfully solved this issue.

Previous studies have applied PT navigation to specific sequences, such as free-breathing T1-mapping [34], cine [35], [36], 5D flow [26], and fat fraction quantification [37]. This prospective study is the first to validate the clinical feasibility and stability of PT-triggered CMR. We enrolled patients with clinical indications for CMR from a single center, covering a broad spectrum of diseases, including common non-ischemic cardiomyopathies (e.g., HCM, dilated cardiomyopathy, myocarditis, and myocardial amyloidosis), ischemic cardiomyopathy, heart valve disease, and cardiac tumors. As a result, the feasibility and stability of PT-triggered CMR were evaluated across a wide range of clinical conditions. All participants underwent two scans, triggered by both PT and ECG, respectively, using the same routine sequences with consistent scanning parameters, allowing each participant to serve as their own control. This design effectively minimized the influence of inter-subject variability and covariates, which are commonly encountered in parallel-group study designs [38].

Lin *et al.* recruited 16 healthy volunteers for a non-contrast CMR study and found that PT-triggered MRI at 1.5T provided comparable measurements of cardiac function, motion, and structure to ECG-triggered CMR [39]. However, this study did not evaluate interobserver variability or contrast-enhanced CMR images, including LGE, post-T1-mapping images, and ECV maps. Moreover, it did not include patients with cardiovascular diseases, whose conditions could potentially affect the reliability of ECG gating. Similarly, Pan *et al.* compared PT- and ECG-triggered non-contrast CMR images in 15 healthy volunteers at two sites using 1.5T and 3T MRI systems. Additionally, only six patients were included for a comparison of PT- and ECG-triggered contrast mapping and LGE images, without cine or T2WI images [40]. In contrast, our study recruited a larger sample of 50 participants, including 15 participants with no apparent cardiac abnormalities on CMR and 35 patients with a wide range of cardiovascular diseases. These conditions encompassed common non-ischemic cardiomyopathies (e.g., HCM, dilated cardiomyopathy, myocarditis, noncompaction of the ventricular myocardium, hypertensive cardiomyopathy, and myocardial amyloidosis), ischemic cardiomyopathy, heart valve disease, cardiac dilatation, pericardial effusion, and ventricular lipoma. Forty-seven participants underwent gadolinium-enhanced CMR (including all patients), while three healthy participants underwent non-contrast scans.

The cardiac trigger detected by the PT system is delayed by approximately 180 ms relative to the R-wave detected by ECG triggering. This delay arises from the PT detecting mechanical motion during the acceleration phase of cardiac contraction and the subsequent low-pass filter processing applied to stabilize the signal [41]. Importantly, this delay is automatically compensated for by the scanner software: when switching from ECG to PT triggering, the system reduces the trigger delay accordingly, except in the case of the dark-blood T2WI sequence, where the fixed timing of the double-inversion preparation makes automatic compensation less feasible. For the TSE dark-blood T2WI sequences, the delayed trigger time of PT may cause slice misregistration between the double-inversion pulse and the excitation pulse, potentially degrading image quality. Although this issue can be mitigated by optimizing acquisition parameters, including the acquisition window, trigger count, trigger delay, and repetition time, no such optimization was applied in the current study. We also observed a slightly higher native T1 values observed in the interventricular septum compared to the lateral wall may be attributed to several factors, including the greater anatomical thickness of the septum, preferential signal reception in the septal region by anterior body coils, and partial volume effects in the lateral wall due to its proximity to lung tissue [42], [43]. While our findings were generally consistent with those of Lin and Pan, we observed that the compCNR of PT-triggered LGE images was higher than that of ECG-triggered LGE images. This may be attributed to the fact that 33 out of 47 contrast-enhanced participants had no visible LGE, and their compCNR values were calculated using blood-myocardium contrast, which is more sensitive to post-contrast timing. Since PT-triggered LGE scans were acquired earlier than ECG-triggered scans, this likely led to higher signal intensities in both myocardium and blood pool. In addition, although post-contrast T1 mapping was acquired earlier for PT-triggered scans compared to ECG-triggered scans, the ECV calculation compensates for such timing differences by incorporating both myocardial and blood pool T1 values. This likely explains the minimal bias observed between PT and ECG-triggered ECV measurements, as shown in Fig. 2**I**. Finally, all image-derived metrics, such as the T2WI SI_myo_/SI_SM_, and compSNR/CNR values from cine and LGE images, may be influenced by factors including parallel imaging, surface coil normalization, and vendor-specific reconstruction algorithms. These values should therefore be regarded as semi-quantitative indicators suitable for intra-individual comparisons under identical acquisition conditions, rather than absolute physical measurements.

The presence of cardiovascular conditions may affect the reliability of ECG gating and image quality [39]. Six participants (No. 005: heart valve disease with atrial dilation; No. 007: obstructive HCM; No. 018: apical HCM; No. 022: obstructive HCM; No. 039: heart valve disease with left atrial dilation; No. 049: obstructive HCM) experienced distortion in ECG signals, leading to incorrect or failed R-wave detection. ECG signals are susceptible to rapid gradient switching and the MHD effect, especially at higher magnetic field strengths [36], and this interference may be exacerbated in the presence of vascular stenoses. The MHD effect within the MRI scanner can distort ECG signals by inducing voltage across blood vessels, which are perpendicular to the static magnetic field, thus complicating R-wave detection. In contrast, PT signals are primarily affected by RF pulses through gain fluctuations in the MR receive chain, which may arise from RF-induced heating of electronic components or from switching of the tune-detune circuit during RF transmission [44]. In fact, PT and ECG are quite complementary in terms of signal detection robustness. While ECG reliability decreases with increasing B0 field strength, PT becomes more robust. Both methods apply more or less complicated signal processing to eliminate the resulting artifacts and stabilize the trigger detection. As such, PT may serve as an alternative method to mitigate image artifacts caused by ECG distortion. However, while PT triggering yielded artifact-free images in these six participants, this observation was based on a small subgroup and should be interpreted with caution. Moreover, ECG trigger settings could not be adjusted or optimized on our system, potentially limiting ECG performance in these cases. In addition, although the PT system operates at low power and poses no additional safety concerns, the presence of metallic implants, even when MRI-compatible, could potentially interfere with signal propagation or reception. While we did not observe any such interference in this study, further investigation is needed to determine whether metallic materials may affect the accuracy or stability of PT-based triggering.

## 5 Limitations

This study has several limitations. First, although the sample size is relatively large and covers a broad range of common cardiovascular diseases, it was conducted at a single center, and only six patients in our cohort exhibited incorrect ECG triggering. Future studies with larger sample sizes and multi-center designs are needed to further validate the findings. Second, although PT triggering functioned reliably in all participants, it is possible that reduced cardiac motion in patients with large body size or impaired left ventricular function could weaken the motion-induced PT signal. Future studies are needed to evaluate PT performance in these specific subgroups. Third, while we aimed to evaluate whether PT could improve image acquisition efficiency in patients with arrhythmia, most arrhythmia episodes were intermittent. As a result, some PT-triggered images were acquired during arrhythmic episodes, whereas the corresponding ECG-triggered images may be obtained during sinus rhythm (or vice versa). This discrepancy made it difficult to perform paired studies. Finally, perfusion imaging was not included in this study. Comparing PT and ECG-triggered perfusion imaging would likely require two contrast injections per patient, which could raise ethical concerns. Additionally, the order of imaging may affect perfusion parameters, further complicating such comparisons.

## 6 Conclusion

Among healthy participants and individuals with common cardiovascular diseases, image quality and most quantitative parameters were comparable between PT-triggered and ECG-triggered conventional sequences. PT may serve as an alternative method to avoid image artifacts caused by ECG distortion, particularly in diseases such as HCM and heart valve disease, where rapid gradient switching and the MHD effect can introduce distortions. In conclusion, PT-triggered CMR represent an effective and promising tool for clinical use.

## Author contributions

**Xianling Qian:** Writing – original draft, Project administration, Methodology, Investigation, Formal analysis, Data curation, Conceptualization. **Yali Wu:** Writing – original draft, Project administration, Methodology, Investigation, Formal analysis, Data curation, Conceptualization. **Peter Speier:** Software, Resources, Methodology. **Caixia Fu:** Software, Resources, Methodology. **Yunzhu Wu:** Software, Resources, Methodology. **Lude Cheng:** Software, Resources, Methodology. **Yinyin Chen:** Supervision, Funding acquisition, Conceptualization. **Shiyu Wang:** Conceptualization. **Caizhong Chen:** Investigation, Data curation. **Kai Liu:** Methodology, Data curation. **Ling Chen:** Formal analysis. **Hang Jin:** Writing – review & editing, Validation, Supervision, Resources, Project administration, Investigation, Funding acquisition, Conceptualization. **Mengsu Zeng:** Writing – review & editing, Validation, Supervision, Resources, Project administration, Investigation, Funding acquisition, Conceptualization.

## Declaration of competing interests

All authors confirm that this paper is solely under submission to *Journal of Cardiovascular Magnetic Resonance* and has not been previously published, in part or whole. No subject overlaps with previously published works. Regarding conflicts of interest, authors P.S., C.X.F., Y.Z.W., and L.D.C. are employees of Siemens healthineers, the manufacturers of the 3.0T MRI equipment used in this study, and have provided advice on sequence parameter adjustments. The remaining authors, not affiliated with Siemens healthineers, controlled and analyzed over all data analysis to mitigate potential conflicts of interest. No other conflicts of interest are declared by the authors.
