# Supplementary material for: Comparison of pilot tone-triggered and electrocardiogram-triggered cardiac magnetic resonance imaging: a prospective clinical feasibility study
Source: J Cardiovasc Magn Reson. 2025 Jun 19;27(2):101925. doi: 10.1016/j.jocmr.2025.101925 (PMC12445410; doi:10.1016/j.jocmr.2025.101925)
Supplement: Supplementary file 1 — Supplementary material [file mmc1.docx]

**Supplemental Materials**

**Table S1:** Consistency of image quality between Reader1 and Reader 2 for PT- and ECG-triggered images

| Sequences | SAX sections | PT | | | ECG | | |
| --- | --- | --- | --- | --- | --- | --- | --- |
|  |  | R1 | R2 | ICC | R1 | R2 | ICC |
| T2WI | Basal | 4 (3 ~ 5) | 4 (3 ~ 5) | .940 | 5 (4 ~ 5) | 5 (4 ~ 5) | .959 |
|  | Mid | 4 (4 ~ 5) | 4 (4 ~ 5) | .827 | 5 (4 ~ 5) | 5 (4 ~ 5) | .947 |
|  | Apical | 5 (4.25 ~ 5) | 5 (4.25 ~ 5) | .864 | 5 (4 ~ 5) | 5 (4 ~ 5) | .943 |
| Native T1-mapping | Basal | 5 (5 ~ 5) | 5 (5 ~ 5) | .789 | 5 (5 ~ 5) | 5 (5 ~ 5) | 1.000 |
|  | Mid | 5 (5 ~ 5) | 5 (5 ~ 5) | .918 | 5 (5 ~ 5) | 5 (5 ~ 5) | 1.000 |
|  | Apical | 5 (5 ~ 5) | 5 (5 ~ 5) | 1.000 | 5 (5 ~ 5) | 5 (5 ~ 5) | 1.000 |
| T2-mapping | Basal | 5 (5 ~ 5) | 5 (5 ~ 5) | / | 5 (5 ~ 5) | 5 (5 ~ 5) | / |
|  | Mid | 5 (5 ~ 5) | 5 (5 ~ 5) | / | 5 (5 ~ 5) | 5 (5 ~ 5) | .781 |
|  | Apical | 5 (5 ~ 5) | 5 (5 ~ 5) | 1.000 | 5 (5 ~ 5) | 5 (5 ~ 5) | / |
| Cine | Basal | 5 (4 ~ 5) | 5 (4 ~ 5) | .950 | 5 (4 ~ 5) | 5 (4 ~ 5) | .902 |
|  | Mid | 4 (4 ~ 5) | 4.5 (4 ~ 5) | .943 | 4.5 (4 ~ 5) | 4.5 (4 ~ 5) | .953 |
|  | Apical | 5 (4 ~ 5) | 5 (4 ~ 5) | .941 | 5 (4 ~ 5) | 5 (4 ~ 5) | .936 |
| LGE | Basal | 5 (4 ~ 5) | 5 (4 ~ 5) | .839 | 5 (4 ~ 5) | 5 (4 ~ 5) | .874 |
|  | Mid | 5 (4 ~ 5) | 5 (4 ~ 5) | .829 | 5 (4 ~ 5) | 5 (4 ~ 5) | .841 |
|  | Apical | 5 (5 ~ 5) | 5 (4 ~ 5) | .807 | 5 (5 ~ 5) | 5 (4 ~ 5) | .824 |
| Post T1-mapping | Basal | 5 (5 ~ 5) | 5 (5 ~ 5) | 1.000 | 5 (5 ~ 5) | 5 (5 ~ 5) | 1.000 |
|  | Mid | 5 (5 ~ 5) | 5 (5 ~ 5) | 1.000 | 5 (5 ~ 5) | 5 (5 ~ 5) | 1.000 |
|  | Apical | 5 (5 ~ 5) | 5 (5 ~ 5) | 1.000 | 5 (5 ~ 5) | 5 (5 ~ 5) | 1.000 |

The ICC value of 1.000 may reflect the limited granularity of the 5-point Likert scale rather than true perfect agreement.

**Note:** PT = Pilot Tone, ECG = electrocardiogram, SAX = short axis, R1 = reader 1, R2 = reader 2, ICC = intraclass correlation coefficient, T2WI = T2-weighted imaging, LGE = late gadolinium enhancement.

**Table S2:** Intraclass correlation coefficient of quantitative assessment between Reader 1 and Reader 2 of PT-triggered images

| AHA 16-segment model | T2WI SI_myo_/SI_SM_ | Native T1-mapping | T2-mapping | Cine compSNR_blood_ | Cine compSNR_myo_ | Cine compCNR | LGE compSNR | LGE compCNR | ECV |
| --- | --- | --- | --- | --- | --- | --- | --- | --- | --- |
| 1 | .993 | .999 | .999 | .993 | .983 | .994 | .986 | .983 | .998 |
| 2 | .994 | 1.000 | .999 |  |  |  |  |  | .998 |
| 3 | .997 | 1.000 | .999 |  |  |  |  |  | .997 |
| 4 | .996 | .999 | .999 |  |  |  |  |  | 1.000 |
| 5 | .986 | .999 | 1.000 |  |  |  |  |  | .999 |
| 6 | .995 | 1.000 | 1.000 |  |  |  |  |  | .999 |
| 7 | .986 | .999 | .999 |  |  |  |  |  | .999 |
| 8 | .984 | 1.000 | 1.000 |  |  |  |  |  | .998 |
| 9 | .992 | 1.000 | 1.000 |  |  |  |  |  | .999 |
| 10 | .994 | .999 | .999 |  |  |  |  |  | .999 |
| 11 | .996 | 1.000 | 1.000 |  |  |  |  |  | .998 |
| 12 | .995 | .999 | 1.000 |  |  |  |  |  | .998 |
| 13 | .992 | .999 | .999 |  |  |  |  |  | .999 |
| 14 | .990 | .998 | 1.000 |  |  |  |  |  | 1.000 |
| 15 | .963 | .999 | .999 |  |  |  |  |  | 1.000 |
| 16 | .988 | .997 | 1.000 |  |  |  |  |  | 1.000 |
| Global | .997 | 1.000 | 1.000 |  |  |  |  |  | .999 |

**Note:** PT = Pilot Tone, AHA = American Heart Association, T2WI = T2-weighted imaging, SI = signal intensity, myo = myocardium, SM = skeletal muscle, compSNR = comparative signal-to-noise ratio, compCNR = comparative contrast-to-noise ratio, LGE = late gadolinium enhancement, ECV = extracellular volume.

**Table S3:** Intraclass correlation coefficient of quantitative assessment between Reader 1 and Reader 2 of ECG-triggered images

| AHA 16-segment model | T2WI SI_myo_/SI_SM_ | Native T1-mapping | T2-mapping | Cine compSNR_blood_ | Cine compSNR_myo_ | Cine compCNR | LGE compSNR | LGE compCNR | ECV |
| --- | --- | --- | --- | --- | --- | --- | --- | --- | --- |
| 1 | .989 | .999 | 1.000 | .992 | .988 | .994 | .982 | .983 | .999 |
| 2 | .990 | 1.000 | .999 |  |  |  |  |  | .997 |
| 3 | .995 | .999 | .999 |  |  |  |  |  | .997 |
| 4 | .989 | .994 | 1.000 |  |  |  |  |  | .998 |
| 5 | .986 | .999 | 1.000 |  |  |  |  |  | .998 |
| 6 | .993 | 1.000 | .999 |  |  |  |  |  | .998 |
| 7 | .997 | .999 | .999 |  |  |  |  |  | .998 |
| 8 | .987 | 1.000 | 1.000 |  |  |  |  |  | .997 |
| 9 | .991 | 1.000 | 1.000 |  |  |  |  |  | .999 |
| 10 | .993 | .999 | 1.000 |  |  |  |  |  | .998 |
| 11 | .991 | .999 | 1.000 |  |  |  |  |  | .998 |
| 12 | .994 | 1.000 | 1.000 |  |  |  |  |  | .998 |
| 13 | .996 | .998 | .993 |  |  |  |  |  | .995 |
| 14 | .991 | .997 | .991 |  |  |  |  |  | .998 |
| 15 | .990 | .998 | .953 |  |  |  |  |  | .993 |
| 16 | .985 | .998 | .663 |  |  |  |  |  | .997 |
| Global | .997 | .999 | .997 |  |  |  |  |  | .999 |

**Note:** ECG = electrocardiogram, AHA = American Heart Association, T2WI = T2-weighted imaging, SI = signal intensity, myo = myocardium, SM = skeletal muscle, compSNR = comparative signal-to-noise ratio, CNR = comparative contrast-to-noise ratio, LGE = late gadolinium enhancement, ECV = extracellular volume.

**Table S4:** Consistency of quantitative assessment of cardiac function and LGE mass between Reader 1 and Reader 2 of PT- and ECG-triggered cine and LGE images

|  | ICC_PT_-R1-R2 | ICC_ECG_-R1-R2 |
| --- | --- | --- |
| Cine-LV EDV | 1.000 | 1.000 |
| Cine-LV ESV | 1.000 | 1.000 |
| Cine-LV SV | 1.000 | 1.000 |
| Cine-LV EF | 1.000 | 1.000 |
| Cine-LV CO | 1.000 | 1.000 |
| Cine-RV EDV | 1.000 | 1.000 |
| Cine-RV ESV | 1.000 | .999 |
| Cine-RV SV | 1.000 | 1.000 |
| Cine-RV EF | .999 | 1.000 |
| Cine-RV CO | 1.000 | 1.000 |
| LGE-mass | .996 | .999 |

**Note:** LGE = late gadolinium enhancement, PT = Pilot Tone, ECG = electrocardiogram, ICC = intraclass correlation coefficient, R1 = reader 1, R2 = reader 2, LV = left ventricular, EDV = end-diastolic volume, ESV = end-systolic volume, SV = stroke volume, EF = ejection fraction, CO = cardiac output, RV = right ventricular.

**Table S5:** Consistency of quantitative assessment of cardiac function and LGE mass between PT-triggered images and ECG-triggered images

|  | PT | ECG | ICC-PT-ECG | *p*-value |
| --- | --- | --- | --- | --- |
| Cine-LV EDV (mL) | 137.87 (110.19 ~ 164.98) | 136.00 (108.81 ~ 164.16) | 1.000 | .198 |
| Cine-LV ESV (mL) | 60.67 (43.91 ~ 86.00) | 59.78 (43.65 ~ 86.94) | 1.000 | .236 |
| Cine-LV SV (mL) | 68.75 (54.82 ~ 84.20) | 67.78 (53.94 ~ 84.03) | .998 | .052 |
| Cine-LV EF (%) | 56.48 (47.00 ~ 61.05) | 57.75 (44.70 ~ 61.31) | .995 | .138 |
| Cine-LV CO (L/min) | 4.80 (3.83 ~ 6.11) | 4.78 (3.91 ~ 6.13) | .985 | .473 |
| Cine-RV EDV (mL) | 125.17 (108.67 ~ 148.68) | 123.50 (106.84 ~ 148.60) | .999 | .188 |
| Cine-RV ESV (mL) | 65.61 (49.59 ~ 92.77) | 65.03 (50.32 ~ 91.66) | .999 | .221 |
| Cine-RV SV (mL) | 61.74 ± 20.20 | 59.22 (46.82 ~ 74.56) | .996 | .800 |
| Cine-RV EF (%) | 47.22 ± 12.35 | 47.70 ± 12.48 | .993 | .148 |
| Cine-RV CO (L/min) | 4.14 (3.19 ~ 5.60) | 4.18 (3.47 ~ 5.66) | .986 | .218 |
| LGE-mass (g) | 10.91 ± 8.98 | 10.80 ± 9.14 | .999 | .397 |

For non-normally distributed data, the values are presented as the median with the interquartile range in parentheses. For normally distributed data, the values are presented as the mean ± standard deviation.

**Note:** LGE = late gadolinium enhancement, PT = Pilot Tone, ECG = electrocardiogram, ICC = intraclass correlation coefficient, LV = left ventricular, EDV = end-diastolic volume, ESV = end-systolic volume, SV = stroke volume, EF = ejection fraction, CO = cardiac output, RV = right ventricular.

**
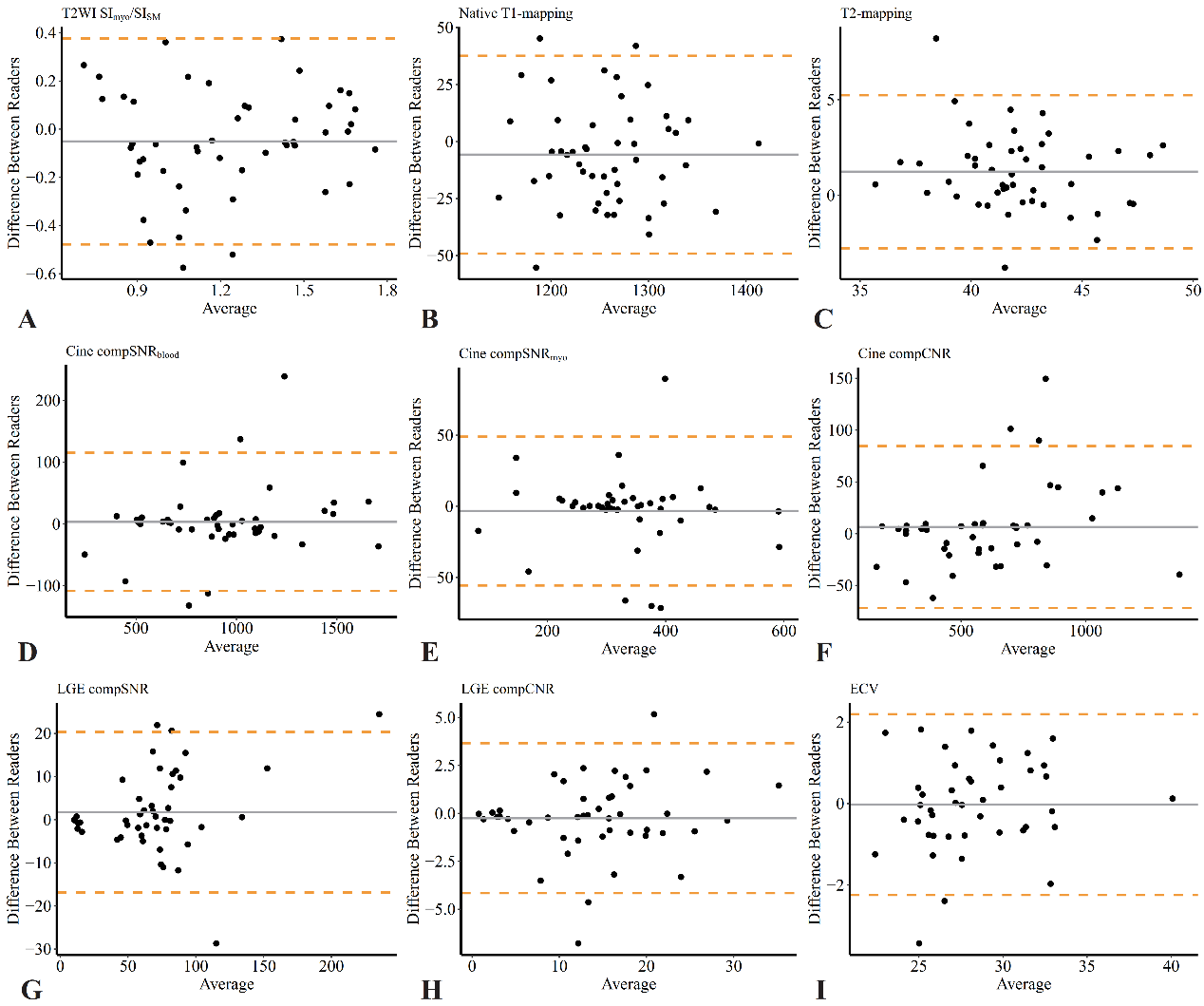
**

**Figure S1:** Bland-Altman plots illustrate the agreement between two readers regarding the parameters of Pilot Tone (PT)-triggered images. These parameters include the ratio of T2WI signal intensity of myocardium to skeletal muscle (SI_myo_/SI_SM_), native T1-mapping value, T2-mapping value, comparative signal-to-noise ratio (compSNR) and comparative contrast-to-noise ratio (compCNR) for cine and late gadolinium enhancement (LGE) images, as well as extracellular volume (ECV).


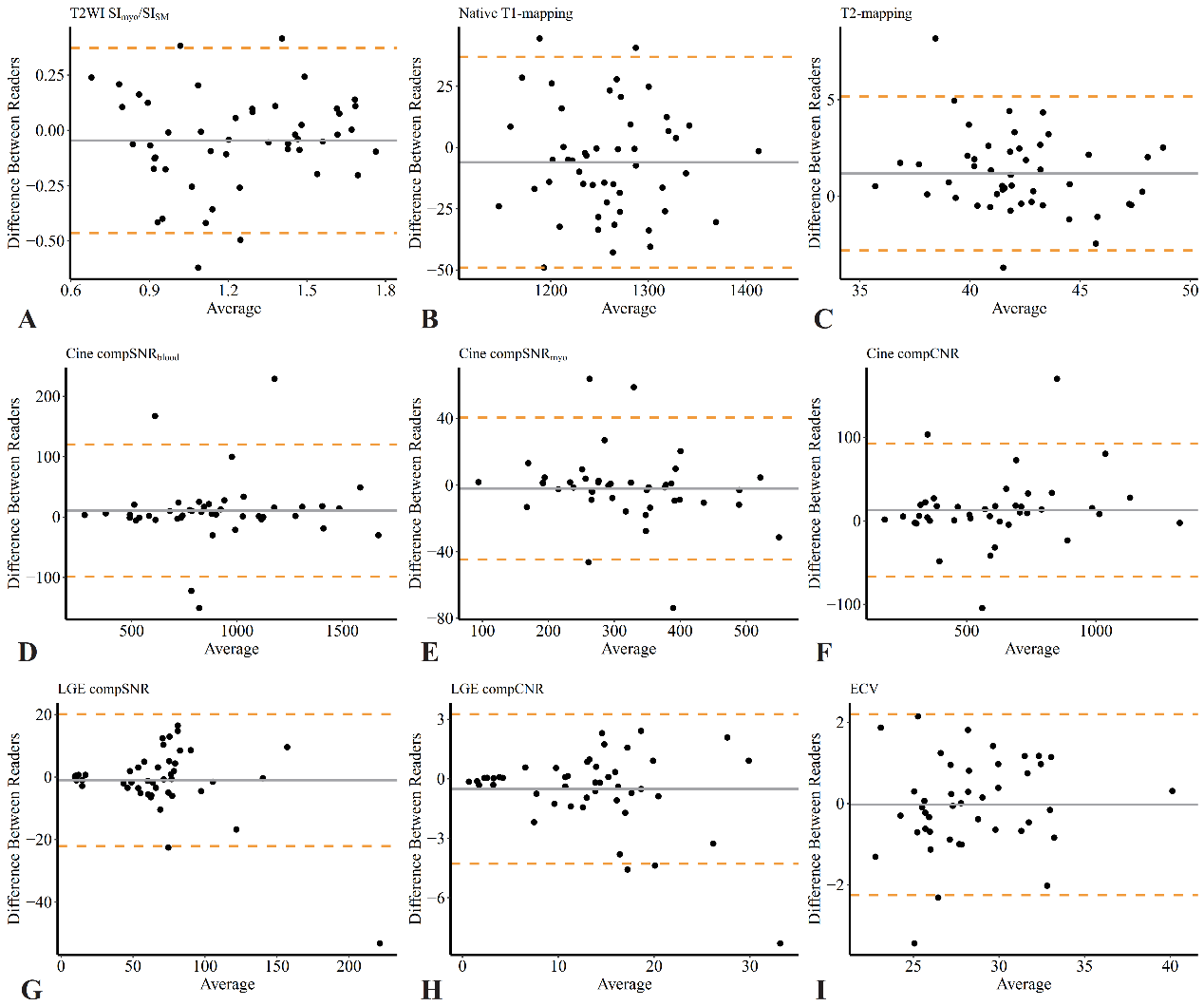


**Figure S2:** Bland-Altman plots illustrate the agreement between two readers regarding the parameters of ECG-triggered images. These parameters include the ratio of T2WI signal intensity of myocardium to skeletal muscle (SI_myo_/SI_SM_), native T1-mapping value, T2-mapping value, comparative signal-to-noise ratio (compSNR) and comparative contrast-to-noise ratio (compCNR) for cine and late gadolinium enhancement (LGE) images, as well as extracellular volume (ECV).
